# Measurement of ship-generated waves in German coastal waterways from 1998–2022

**DOI:** 10.1038/s41597-024-04299-5

**Published:** 2025-01-11

**Authors:** Arne Seemann, Gregor Melling

**Affiliations:** Federal Waterways Engineering and Research Institute, Department of Coastal Engineering, Hamburg, Germany

**Keywords:** Environmental impact, Physical oceanography

## Abstract

Vessel-generated waves and currents significantly impact coastal and estuarine waterways. *In-situ* measurements record all relevant physical phenomena that occur under a wide range of conditions and are therefore a valuable resource in the investigation of ship waves. Here we present a comprehensive compound dataset from *in-situ* ship wave measurement campaigns conducted over several decades in German coastal waterways. The dataset includes measured ship waves heights and currents as well as an attribution of each wave event to ship and nautical parameters responsible for its generation. The dataset is unique in its scope, combining a significant number of data points from multiple sites with different cross-section profiles. Some campaigns feature multiple gauges per cross-section, facilitating the analysis of wave transformation processes. Some locations feature repeat measurements which helps to understand wave load histories due to e.g. changes in shipping fleet structures and navigation practices. This dataset aims to provide a basis to enhance data-driven models to predict and manage the impacts of vessel-generated waves in different topographic settings and fleet structures.

## Background & Summary

**Fig. 1:**
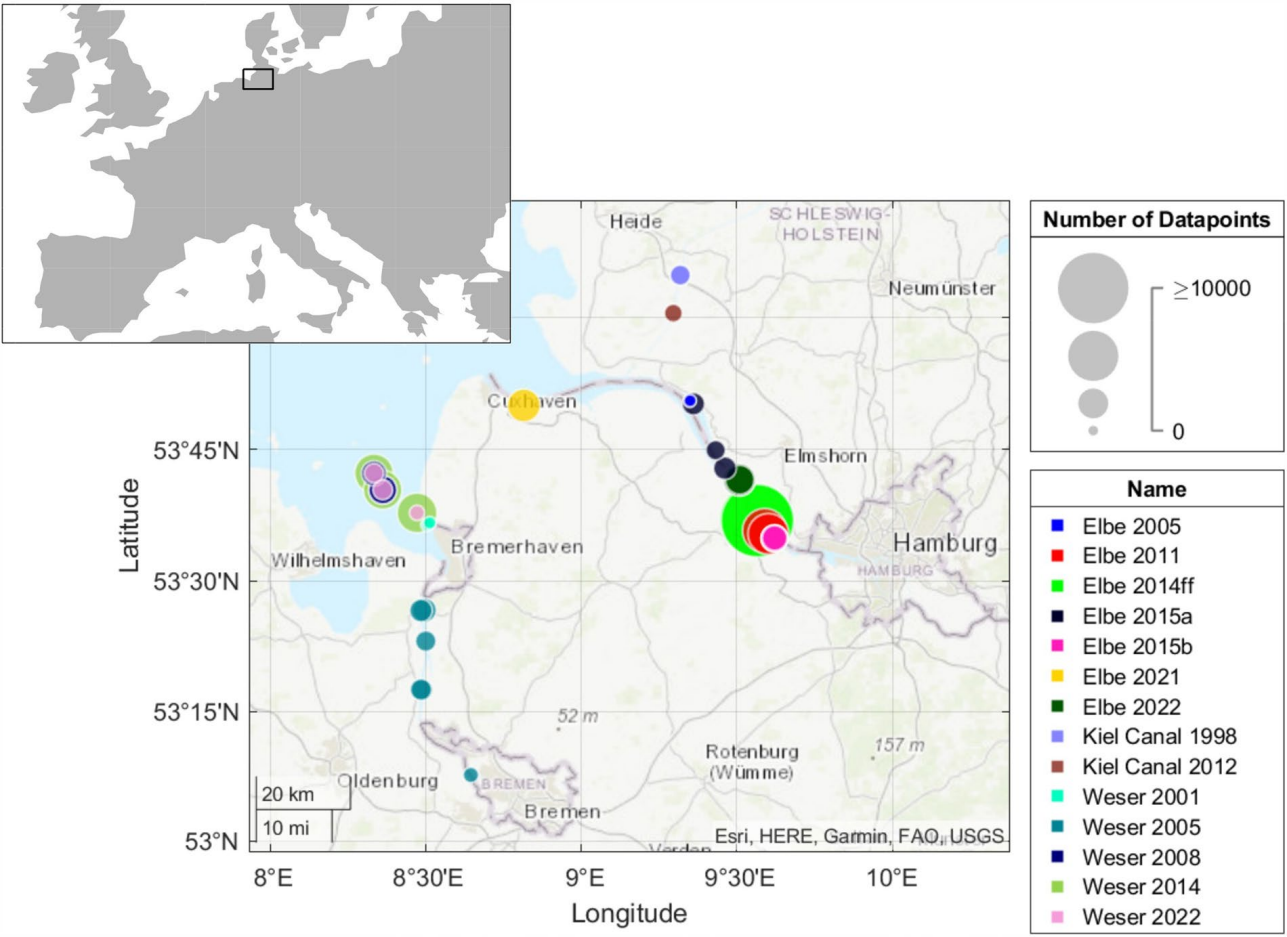
Map of northwest Germany showing measurement sites and number of recorded ship wave events.

Vessel-generated waves and currents are a crucial load factor to the banks of coastal and estuarine waterways^1,2^. In particular the action of the long-period wave components have been implicated in a number of deleterious effects in shallow and confined coastal and estuarine waterways^3^ such as sediment resuspension^4,5^, erosion of banks^6–8^, mudflats^9^ and marshes^10,11^, structural damages^12–14^, and ecological habitat alteration and deterioration^15^. These impacts of ship-induced waves and currents are particularly evident in sheltered bays and estuaries which are not adapted to high-energy loads. In Germany, the largest ports of Hamburg and Bremerhaven are located in estuaries, which in turn border the UNESCO world heritage site Wadden Sea. Both ports are frequented by the largest container ships in the world. Additionally, the Kiel Canal is one of the world’s busiest artificial waterways. This prevalence of loads associated with, in particular, commercial shipping has led to a significant number of *in-situ* measurement campaigns in these waters over the last decades (cf. Figure 1).

**Fig. 2:**
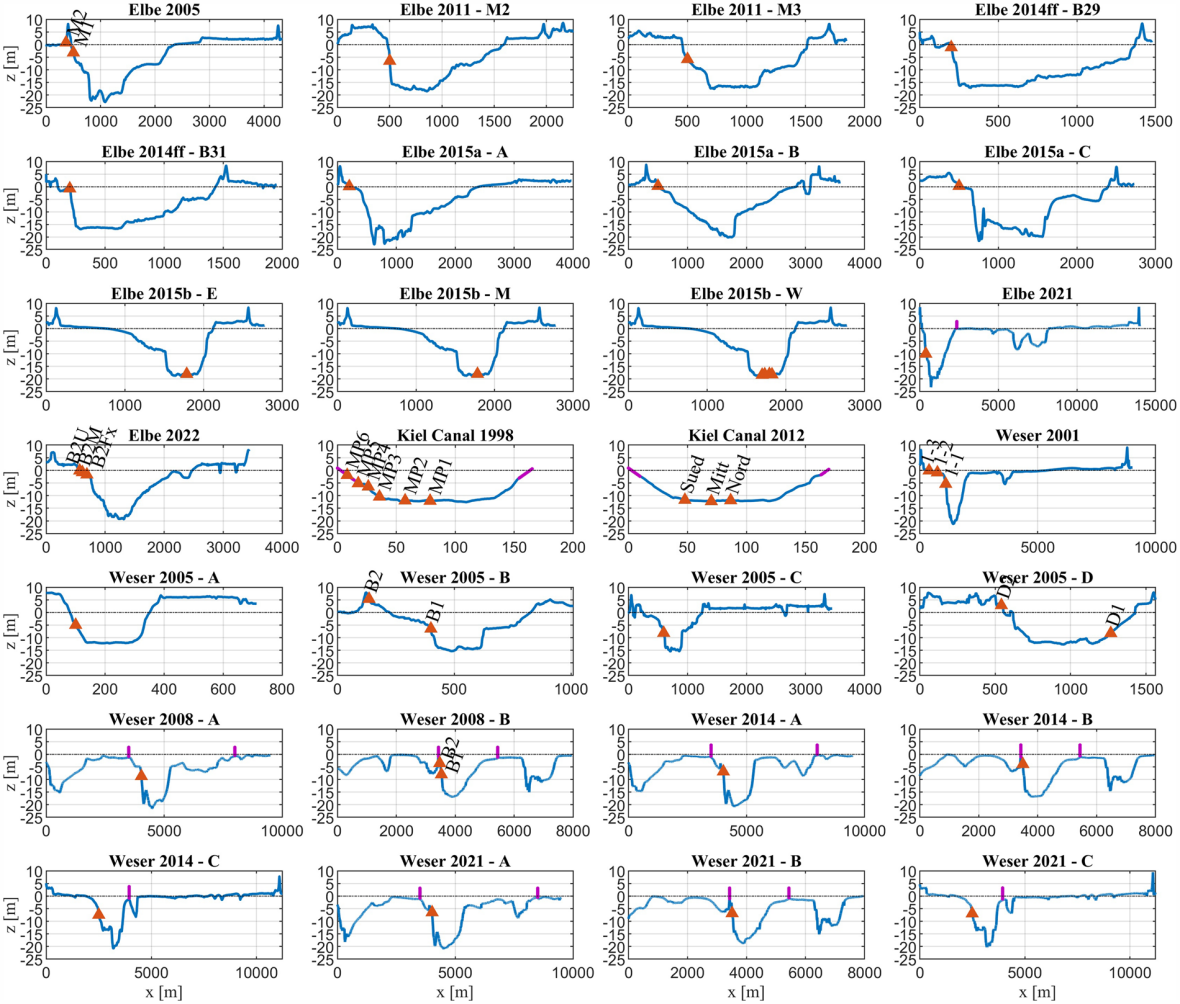
Cross-section geometries of the different measurement campaigns. The red triangles indicate the location of the measurement stations. The pink lines are manually inserted artificial boundaries, which are necessary for the calculation of the cross-section geometry parameters.

**Fig. 3:**
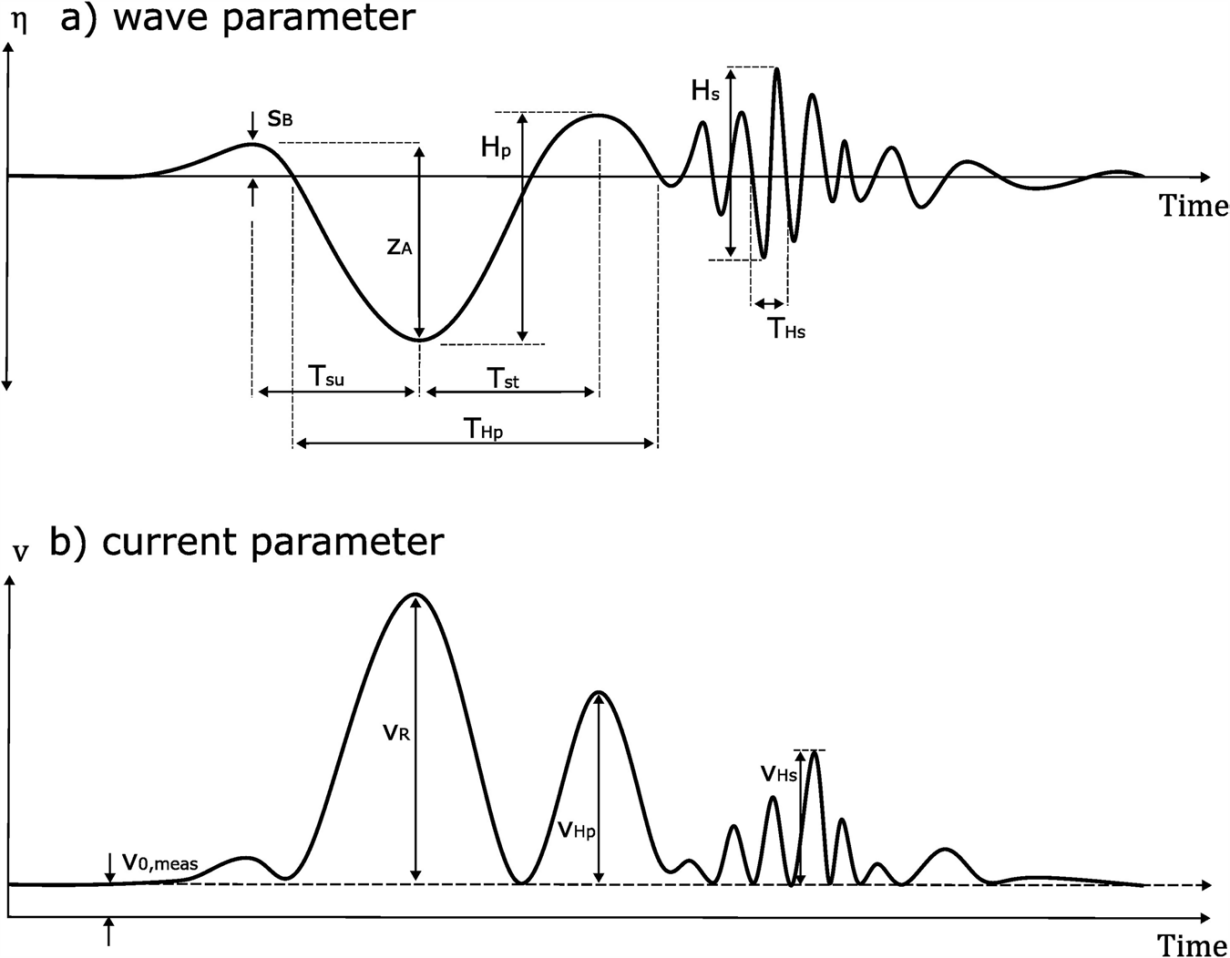
Definition of the ship induced wave and current parameters, where ${s}_{B}$ is the bow wave height, ${z}_{A}$ the drawdown height, ${H}_{p}$ the primary wave height, ${H}_{s}$ the secondary wave height, ${T}_{{su}}$ the time from bow wave to drawdown, ${T}_{{st}}$ is time from drawdown to the maximum primary wave, ${T}_{{Hs}}$ the secondary wave period, ${v}_{0,{meas}}$ the tidal flow velocity at the measurement gauge, ${v}_{R}$ the return current velocity, ${v}_{{Hp}}$ is primary wave current velocity, and ${v}_{{Hs}}$ is the secondary wave current velocity.

The measurements were stored and documented, so that they can be compiled into the dataset presented here. All the measurements are point measurements taken either in or at the edge of the fairway or close to the shore. While a variety of instruments were used in the different campaigns, the required target parameters of water level variations and currents are standardised (cf. Figure 3). Each recorded wave event is assigned to its causing ship. To every campaign, a cross-section bathymetry is assigned and different geometrical parameters are calculated. This allows conclusions to be drawn on the relationship between ship wave loads and vessel size, navigation behaviour, and waterway geometry, a task which is of great interest in research literature and for which numerical^16–18^, physical^19^, or data driven models^20–22^ have been applied. However, the publications on data driven models in this field are mostly based on individual (or small number of) measuring stations, with a limited number of ship passages, and wave parameters narrowing the scope of possible investigations. The dataset at hand is unique in that it: (i) collates a large amount of data points from different localities in a consistent dataset, (ii) offers measurements in different cross-sections at varying depths (Fig. 2), and (iii) includes repeat measurements at some sites. The authors believe that the presented dataset can be used to improve the prediction of vessel-generated waves in varying topographic settings, as well as providing a time-history of wave loads through changes of shipping fleet structure, and navigation practices. Some measurement campaigns feature multiple gauges in one cross-section, allowing analysis of the wave transformation process towards the bank under different conditions.

## Methods

In the following, the general process of the individual measurement campaigns and the compilation into the dataset are laid out. Detailed information for every measurement campaign is summarized in the metadata table, which is provided with the dataset^23^.

### Measurements

The individual measurement campaigns were carried out independently, by different operators, and with different goals in mind. This results in a dataset that offers some heterogeneity when seen in its entirety, yet consistency when singular campaigns are considered. The measurements are arranged at different locations within the waterway cross-sections as seen in Fig. 2 and a varying set of instruments were used depending on conditions, requirements, and purpose of measurement. For waves, in the majority of the campaigns, pile-mounted resistance wave gauges were used. In shallow-water near-bank locations or when an underwater bottom mount was required, pressure gauges were employed. The flow velocity measurements were conducted using electromagnetic current meters or acoustic doppler devices.

### Processing of the measurements

The hydrodynamics of ship-generated waves in sub-critical speed regime can be grouped in a primary and a secondary wave system. The primary wave system (also called Bernoulli wake^24^, depression wake^25^ or low-frequency wake^8^) is generated by the displacement of the ship travelling through a confined waterway. This leads to a return current v_R_ from bow to stern of the vessel and by energy conservation to a water level depression, which is termed drawdown z_A_. As it is bound to the ship, the measured period of the primary wave (as defined in Fig. 3) is significantly longer than e.g. wind waves $(\bar{{T}_{{H}_{P}}}=148{s}$ in the dataset). The secondary waves are caused by pressure gradients at the bow and stern of the ship and consist of wave packets with wave periods similar to local wind waves $(\bar{{T}_{{Hs}}}=3.0{s}$ in the dataset). The fact that both vessel-generated wave systems have certain frequency spectra allows them to be separated using low or high-pass filters. Wind wave heights are determined in periods where no secondary waves occur, as they are not separable by frequency. From the processed wave signals, the characteristic parameters displayed in Fig. 3 were calculated. Not all parameters were determined in all campaigns, however the primary wave height ${H}_{p}$ is available for all stations. In a next step, the wave parameters are correlated to the corresponding ship passage, using AIS data (Automatic Identification System). The AIS data contains static (e.g. length, width) and dynamic (e.g. draught, speed over ground) vessel information. To calculate the relative speed through water ${U}_{{STW}}$ of the ship, which is relevant for wave generation, the flow velocity in the fairway channel has to be determined. Different methods were used to gather this value. Either it was extrapolated from measurements at the fairway edge or near the bank, interpolated from nearby hydrological measurement stations or extracted from operational hydrodynamic models.

For detailed information about each individual measurement campaign the reader is referred to the metadata table.

### Processing of the dataset

In order to simplify and enrich future analyses based on the dataset, several processing steps were carried out by the authors. First, the data of all measurement campaigns was processed into a standardised format and table structure. To ensure data quality and consistency, the data was filtered in multiple steps. In order to be available for later analyses, all data which was removed during the filtering process is stored in a separate file, to be available if necessary. The AIS data used to obtain the vessel information may contain incorrect or missing values. These were identified and filtered using known minimum and maximum values of vessel width and length, typical combinations^26^ of both, and maximum possible draught. The values for these limits are listed in the documentation, which is also attached to the dataset^23^. If unambiguously identifiable, fast ferries were removed, as the wave physics of these ships are not comparable with large displacement vessels travelling at subcritical speeds and cannot be described with the same wave parameters shown in Fig. 3. Wave events where the instrument becomes emersed (i.e. exposed to the air), as can happen when a large drawdown event occurs during low ambient water level, were likewise removed as, in this case, parts of the wave record are missing. When multiple ships cross the measurement section in close temporal proximity, either superimposed waves or incorrect assignments of the waves to the ship can occur. This is particularly erroneous, if smaller ships are assigned a bigger wave of a large ship, and vice versa. A duplicate assignment of the same wave is also possible. In some campaigns these events are identified, marked and removed from the dataset. However, if and how the ship encounters are defined varies between the measurements, which results in a heterogeneity that must be considered.

**Fig. 4:**
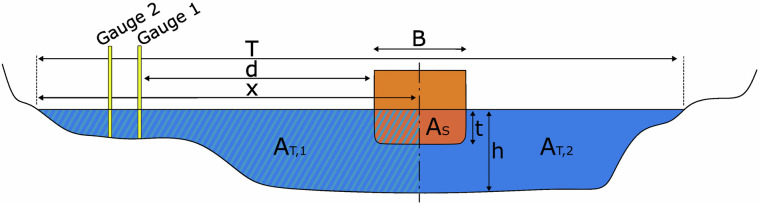
Definition of geometrical cross-section parameters with the submerged midship cross-section ${A}_{S}$, the river cross-sectional area on each side of the ship ${A}_{T,1}$ and ${A}_{T,2}$, the top width of the river T, the passing distance of the ship to the gauge d, the passing distance of the sailing line to the shoreline x, and the ship width B, draught t and water depth h. If multiple gauges are in one cross-section, the passing distance is referenced to the nearest station.

In a next step, bathymetric data, in form of cross-sectional profiles, are assigned to every measurement location. Those were extracted from digital terrain models of the waterway^27^ or from temporal and spatial interpolated yearly terrain models^28^. They were selected to correspond to the year of the measurement. The cross-sections are used to calculate geometric parameters shown in Fig. 4 for every ship passage at every measurement station. For open cross-sections where one or both sides of the fairway are adjacent to a shallow foreshore or tidal flat, it is not possible or practical to calculate these parameters. Therefore, artificial boundaries were inserted at the edge of the fairway. These were either placed on structures such as the training walls (e.g. Weser 2014) or on the embankment shoulder of the fairway (e.g. Elbe 2021). These boundaries are illustrated in Fig. 2. In Kiel Canal 1998, the cross-section was extended with typical bank slopes.

Additionally, several bathymetric parameters are calculated which are expected to correlate with the ship-induced hydrodynamic loads^13,20,29,30^:

- Depth related Froude number: ${F}_{h}=\,\frac{{U}_{{STW}}}{\sqrt{g\bullet h}}$
- Blockage factor: $$n=\frac{{A}_{T,1}{+A}_{T,2}}{{A}_{S}}$$
- Partial Blockage factor of the gauge-facing side: ${n}_{T,1}=\frac{{A}_{T,1}}{{0.5\bullet A}_{S}}$
- Depth to draught ratio: $\frac{h}{t}$
- Partial eccentricity factor: ${m}_{A}=\frac{{A}_{T,1}}{{A}_{T,1}{+A}_{T,2}}$

As mentioned above, the secondary waves and wind waves cannot be separated by frequency. In order to avoid erroneous registration of above-average wind waves as secondary waves, the criterion ${H}_{S} > 2.25\,{H}_{{sig}}$ was applied, where ${H}_{{sig}}$ is the significant wind wave height. All secondary waves below this threshold are omitted. This criterion stems from the relationship between the maximum and the significant wind wave height^30^ ${H}_{\max }\approx 2\,{H}_{{sig}}$ and has proven to be robust in multiple measurements campaigns.

## Data Records

The dataset is publicly available in the data repository^23^ of the German Federal Waterways Engineering and Research Institute (BAW). It contains the quality-assured datasets, the data removed during filtering process, the cross-section bathymetries, the metadata table including detailed information about each measurement campaigns and a dataset documentation, which includes an explanation of the column headers and the applied filter rules. The data is also provided as .mat file in which the ship wave data is stored together with the cross-section data in a struct array.

**Fig. 5:**
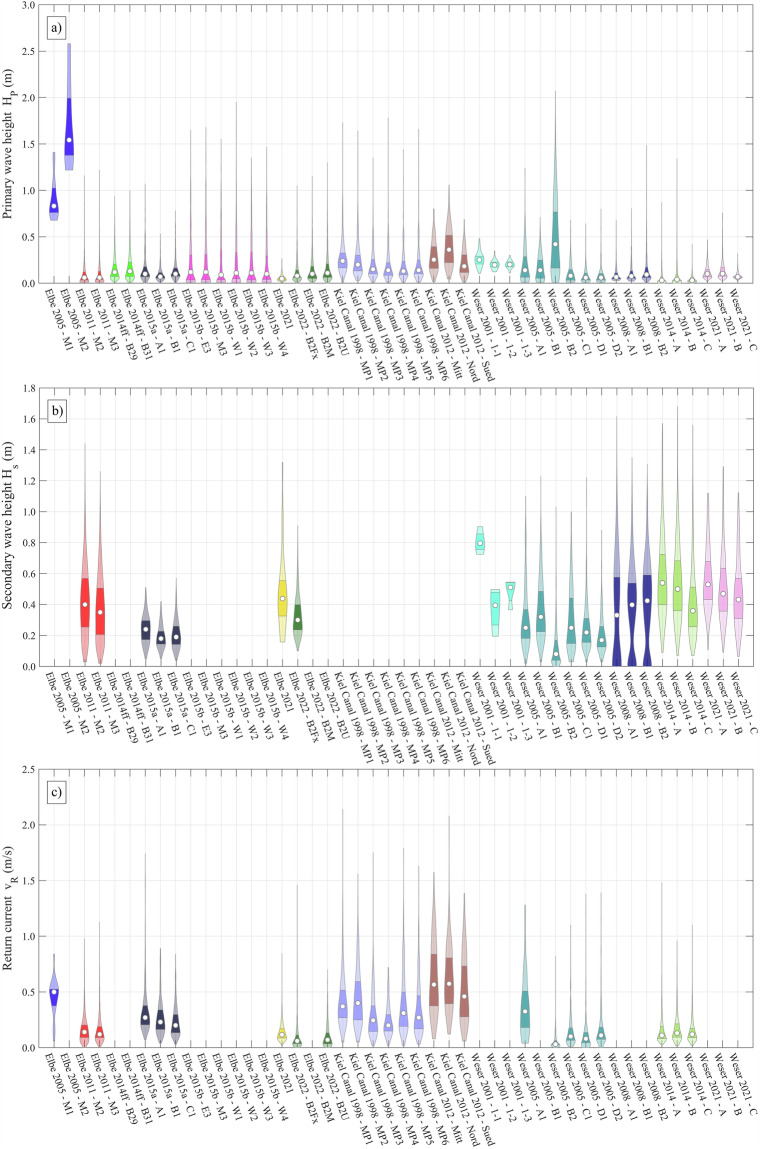
Overview of the wave parameter distribution. The colors correspond to those used in Fig. 1. The distribution is represented as violin plots, which utilizes kernel density estimation. The boundaries of the dark colour areas denote the upper and lower quartiles. The white circle depicts the median. No violin is displayed if values are missing (cf. Table 1).

**Fig. 6:**
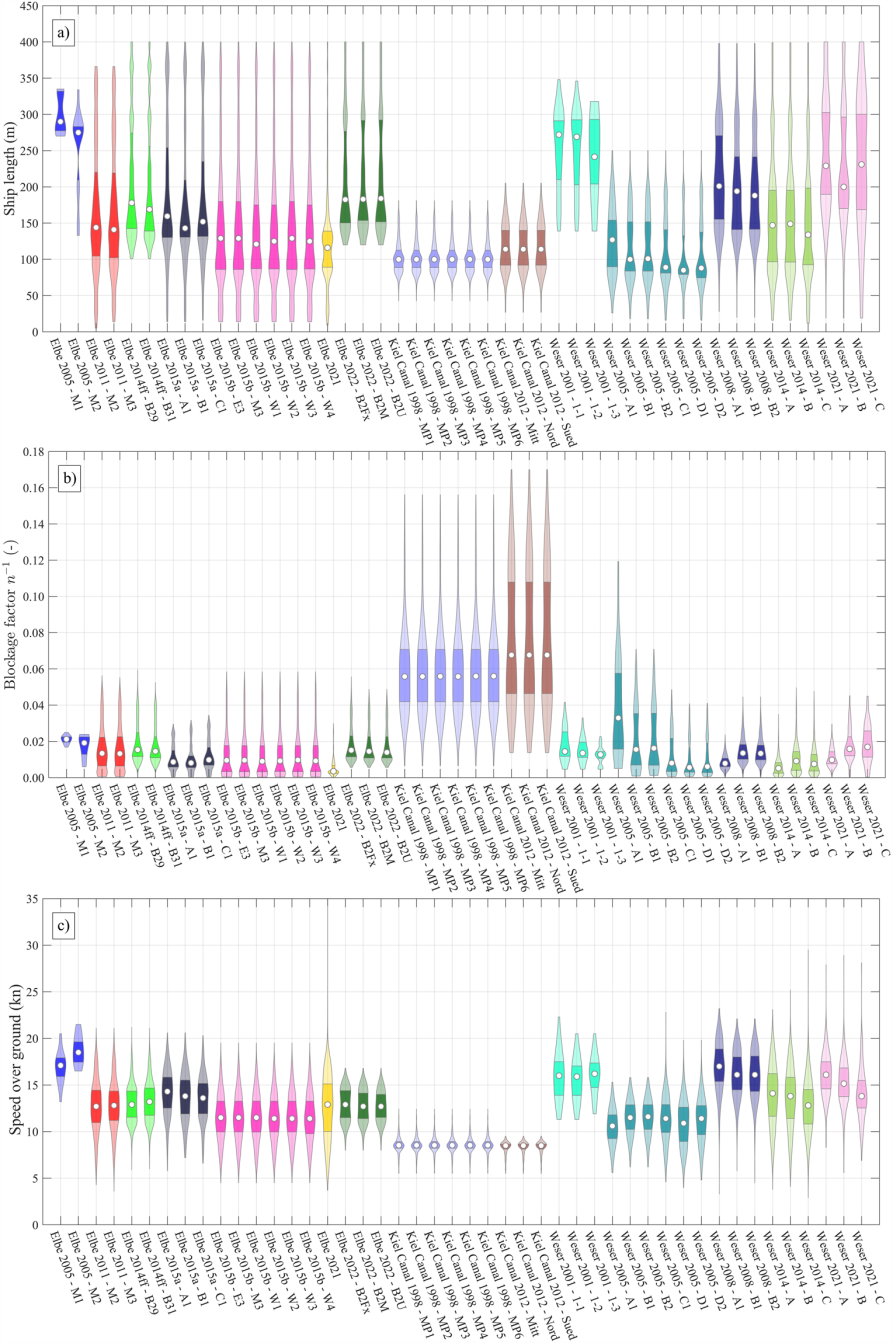
Overview of the vessel parameter distributions. The blockage factor is inversed for intuitive interpretation. The colors correspond to those used in Fig. 1. The distribution is represented as violin plots, which utilizes kernel density estimation. The boundaries of the dark colour areas denote the upper and lower quartiles. The white circle depicts the median. No violin is displayed if values are missing (cf. Table 1).

An overview over the available ship wave measurement campaigns and available data is presented in Table 1. In summary, 97.877 data points before, and 81.092 after the filtering process from 46 measurement stations in 28 different cross-sections in 14 different measurement campaigns are available. Cross-sections from the same location but different years are counted individually as they have slightly different bathymetries. Otherwise, there are 23 different cross-sections, some of which were measured several times. The most frequently measured cross-section is profile B in the Outer Weser, which was instrumented in the Weser 2008, 2014, and 2021 campaigns. The size of the individual datasets varies considerably. One reason for this is the different measurement duration. Another reason is the minimum wave height threshold at which each of the waves were processed. For example, in Weser 2014 waves with ${z}_{A}\ge 1$ cm were registered. In Weser 2021, on the other hand, only waves with ${H}_{p}\ge 5$ cm or ${z}_{A}\ge 5$ cm were recorded, which corresponds only to about 20% of all ship passages. In Elbe 2005, only the 33 highest events were documented. Also, depending on the purpose of the campaign and the available instruments, different wave parameters were evaluated. For example, in campaigns where bottom-mounted pressure gauges were used to measure the primary wave height, no secondary waves were evaluated due to the dampening of the pressure signal in water column. Flow velocity data is available for 26 measurement stations. Figure 5 gives an overview of the measured ship-induced wave parameters and Fig. 6 an overview of the corresponding vessel size and speed and the degree of confinement as given by the blockage factor.

**Table 1:** Information about measurement campaigns and data availability.

The green color means that the parameter is available at all stations, orange at some stations, and red at no stations in the respective measurement campaign.

## Technical Validation

Unfortunately, there are no independent measurements which would allow the direct validation of the measurement campaigns. However, it is possible to show the plausibility and the consistency of the dataset using statistical analysis.

In order to show the validity of wave-to-ship-assignment under consideration of the bathymetric attributes, a regression analysis was performed. For this purpose, Eq. 1 was fitted to every measurement station in the dataset individually.

1 $${H}_{p}={b}_{1}\cdot {n}^{{b}_{2}}\cdot {{F}_{h}}^{{b}_{3}}\cdot {{m}_{A}}^{{b}_{4}}$$

**Fig. 7:**
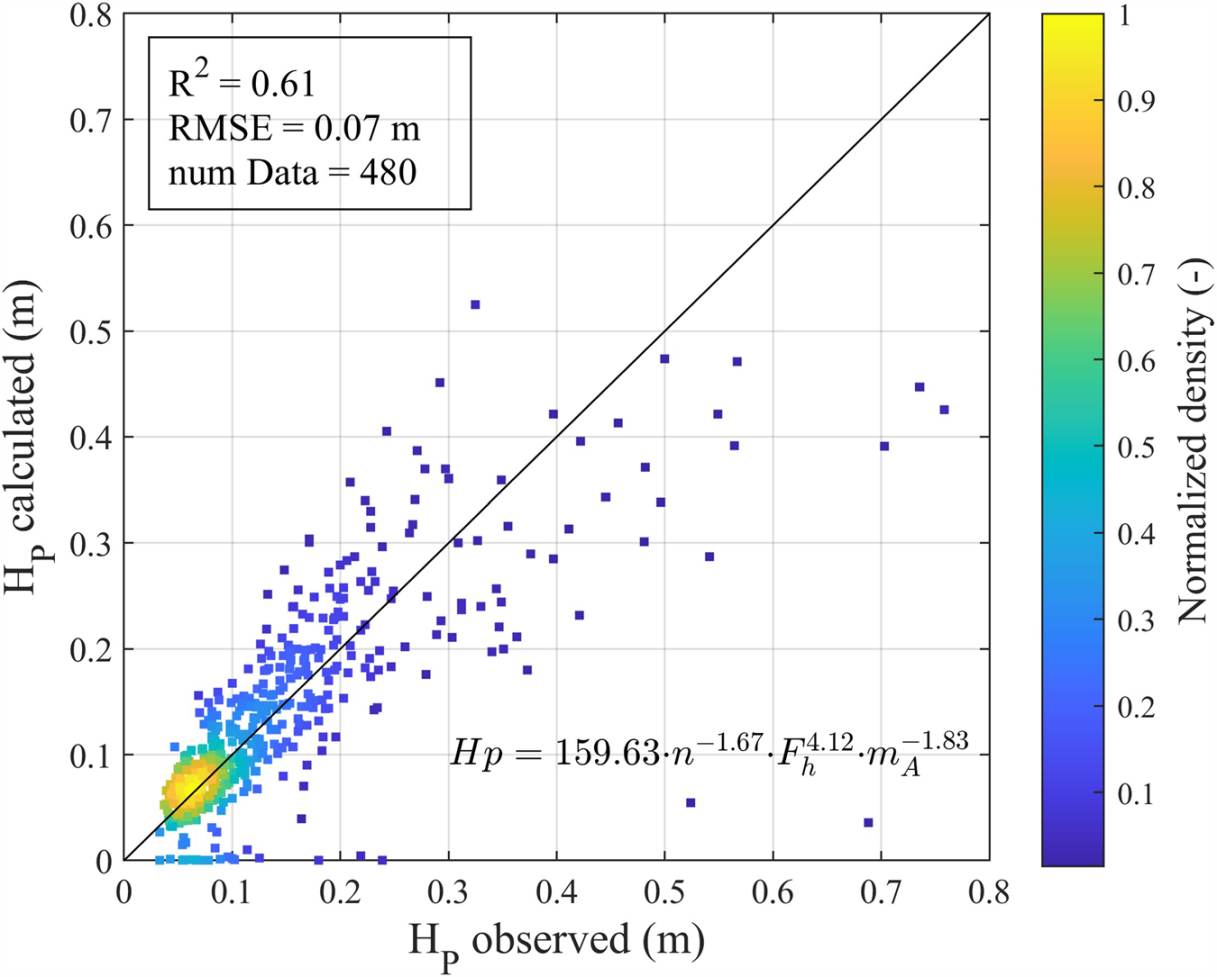
Regression result from Weser 2021 station B.

The equation combines the primary wave height ${H}_{p}$ (in meters) with the blockage factor $n$, which is a function of ship size and cross-section water area, the depth related Froude number ${F}_{h}$, which combines the ship’s speed through water and the water depth, and the partial eccentricity factor ${m}_{A}$. All stations were fitted individually to Eq. 1. An example of the results is shown in Fig. 7 for Weser 2021 Station B. The ${R}^{2}=0.61$ shows a good correlation of the input parameters to the primary wave height. The coefficients of this model can be interpreted in a way that larger ships, faster ships or ships travelling closer to the bank produce larger waves, and vice versa, which corresponds well to the physical understanding.

However, there are also outliers with small calculated and larger observed primary wave height values. These outliers may be incorrectly assigned ships or due to other residual uncertainties inherent in the dataset. Major sources of uncertainty resulting from the measurement and processing of the data are assumed to be e.g.: i) the AIS draught value, which is manually edited by the crew after each destination, ii) the determination of the flow velocity in the fairway, and iii) the definition of ship encounters, which was not considered in all campaigns. Those uncertainties should be taken into account when using the data.

**Fig. 8:**
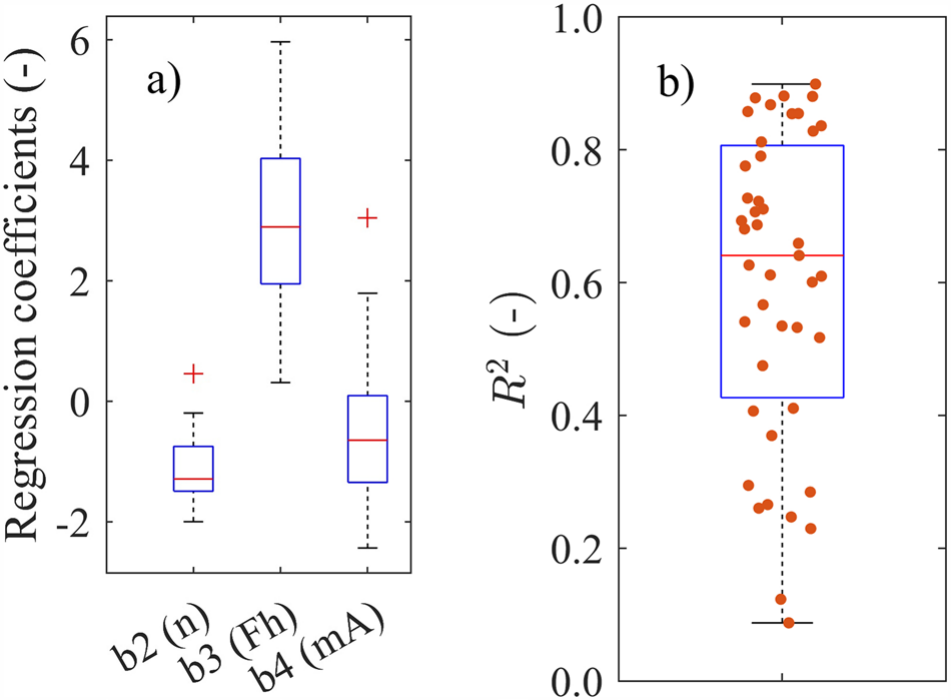
(**a**) Boxplot showing the regression coefficient for all measurement stations fitted to Eq. 1, (**b**) Boxplot showing the distributions of the coefficient of determination ${R}^{2}$ for all measurement stations fitted to the Eq. 1.

Figure 8a shows the distribution of the coefficients ${b}_{2}$ to ${b}_{4}$ for all campaigns fitted individually to Eq. 1. In general, the coefficients follow the same direction, as described above. However, there are exceptions with sign reversal of ${b}_{2}$ and ${b}_{4}$, which in turn represent ship size and passing distance. These exceptions occur in campaigns with few data points (Elbe 2005), with very large passing distances (e.g. Elbe 2021), and when measuring directly in the fairway (e.g. Elbe 2015b). These measurements are also subject to poor ${R}^{2}$ values, which are displayed in Fig. 8b. Beside these outliers this basic model seems to represent the data quite well and thus the data can be considered valid.

## Usage Notes

As described, the authors have performed a data filtering procedure and diligently removed obvious erroneous values. Nevertheless, as shown in Fig. 7, some residual uncertainties surrounding outliers remain. The executed filtering process is meant to remove wave event with missing data (e.g. no draught value), erroneous values (e.g. ship length/width), and unclear ship assignments (encountering ships). However, this means that some waves will be lost from the data set. Depending on the purpose of the data, the filter steps can be adjusted or reversed, as all the data is still available in the dataset. Prospective users are also encouraged to be vigilant to the possibility that some erroneous values are still present in the dataset, to evaluate critically, perform further filtering steps, and outlier analysis as required by their application. If required, further unpublished internal technical reports and additional data (e.g. the raw time series) are available and can be requested from the authors.

## Data Availability

No custom code was used to generate or process the data described in this manuscript.
